# Supplementary material for: Tetrodotoxin and the Geographic Distribution of the Blue-Lined Octopus Hapalochlaena fasciata on the Korean Coast
Source: Toxins (Basel). 2023 Apr 11;15(4):279. doi: 10.3390/toxins15040279 (PMC10145357; doi:10.3390/toxins15040279)
Supplement: Supplementary file 1 [file toxins-15-00279-s001.zip › toxins-2194228-supplementary.pdf]

(A) Standard

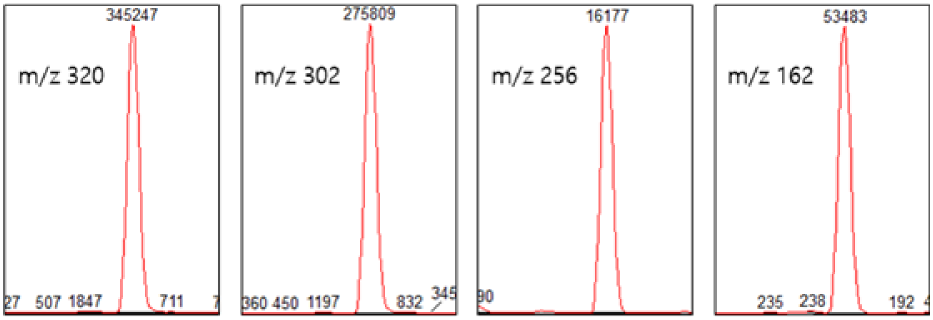

(B) Sample

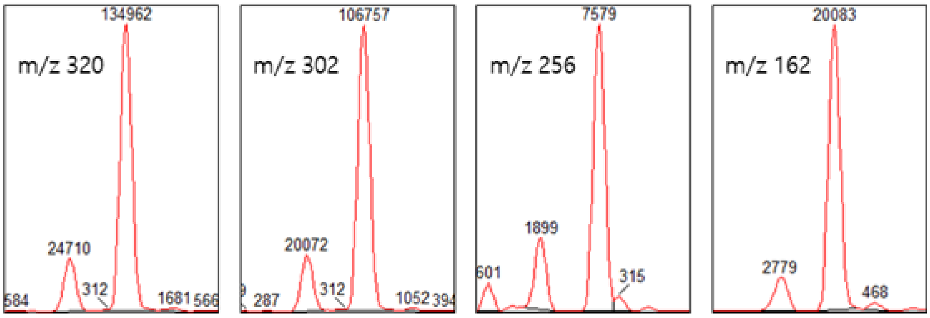

m/z	Peak ratio (%)	
	Standard (A)	Sample (B)
320	100	100
302	79.9	82.8
256	4.7	4.4
162	15.5	12.6

Figure S1. The ratio of the peaks in the TTX standard (A) and tissue sample (B). The table represents the rato of each peak at 320, 302, 256, and 162 *m/z* in the TTX standard (A) and tissue sample (B).
